# Fine mapping and target gene identification of *qSE4*, a QTL for stigma exsertion rate in rice (*Oryza sativa* L.)

**DOI:** 10.3389/fpls.2022.959859

**Published:** 2022-07-18

**Authors:** Naihui Guo, Yakun Wang, Wei Chen, Shengjia Tang, Ruihu An, Xiangjin Wei, Shikai Hu, Shaoqing Tang, Gaoneng Shao, Guiai Jiao, Lihong Xie, Ling Wang, Zhonghua Sheng, Peisong Hu

**Affiliations:** ^1^State Key Laboratory of Rice Biology, Key Laboratory of Rice Biology and Breeding, Ministry of Agriculture, China National Rice Improvement Centre, China National Rice Research Institute, Hangzhou, China; ^2^Rice Research Institute, Shengyang Agricultural University, Shenyang, China

**Keywords:** stigma exsertion rate, QTL, near-isogenic line, hormone, rice

## Abstract

The stigma exsertion rate (SER) is a complex agronomy phenotype controlled by multiple genes and climate and a key trait affecting the efficiency of hybrid rice seed production. Using a japonica two-line male sterile line (DaS) with a high SER as the donor and a tropical japonica rice (D50) with a low SER as the acceptor to construct a near-isogenic line [NIL (*qSE4*^DaS^)]. Populations were segregated into 2,143 individuals of BC_3_F_2_ and BC_4_F_2_, and the stigma exsertion quantitative trait locus (QTL) *qSE4* was determined to be located within 410.4 Kb between markers RM17157 and RM17227 on chromosome 4. Bioinformatic analysis revealed 13 candidate genes in this region. Sequencing and haplotype analysis indicated that the promoter region of *LOC_Os04g43910* (*ARF10*) had a one-base substitution between the two parents. Further Reverse Transcription-Polymerase Chain Reaction (RT-PCR) analysis showed that the expression level of *ARF10* in DaS was significantly higher than in D50. After knocking out *ARF10* in the DaS background, it was found that the SER of *arf10* (the total SER of the *arf10-1* and the *arf10-2* were 62.54 and 66.68%, respectively) was significantly lower than that of the wild type (the total SER was 80.97%). Transcriptome and hormone assay analysis showed that *arf10* had significantly higher auxin synthesis genes and contents than the wild type and the expression of auxin signaling-related genes was significantly different, Similar results were observed for abscisic acid and jasmonic acid. These results indicate that *LOC_Os04g43910* is mostly likely the target gene of *qSE4*, and the study of its gene function is of great significance for understanding the molecular mechanisms of SER and improving the efficiency of hybrid seed production.

## Introduction

Rice (*Oryza sativa* L.) is a staple food for billions of people worldwide (Khush, 2005). High yield has always been the primary goal of rice breeding in China for the past few decades. The successful development of hybrid rice was another milestone in enhancing rice yield following dwarf breeding. Rice is a typical self-pollinating crop (Virmani and Athwal, 1973), but the male sterile line with pollen abortion cannot self-fertilize, so the male sterile line is the key line for hybrid rice breeding. Stigma exsertion is an important agronomic trait for male sterile lines to successfully receive restorers’ pollen. Studies have shown that flowering does not mean fertilization (Kato and Namai, 1987), and the exposed stigma can maintain vigor for 4 to 6 days, which greatly improves the outcrossing rate of rice; thereby improving the seed production efficiency of hybrid rice (Yan and Li, 1987; Bi and Tan, 1988; Tian et al., 2004). Therefore, the stigma exsertion rate (SER) of male sterile lines is a key factor of hybrid rice. Increasing the SER is beneficial to increase the yield of hybrids and promote their commercialization (Virmani, 1994).

In the past two decades, dozens of QTLs related to the SER, distributed on 12 chromosomes of rice, have been detected by various germplasm resources and methods (Miyata et al., 2007; Yan et al., 2009; Liu et al., 2019; Tan et al., 2020). For this research, male sterile lines, maintainer lines, and wild rice with high stigma exsertion are commonly used. From the two-line male sterile line DaS, *qPES3*, *qPDES4,* and *qPES12* were mapped on chromosomes 3, 4, and 12, respectively (Li et al., 2017). Using the single-segment substitution line created by the maintainer line IR66897B, *qSER-2a*, *qSER2b,* and *qSER3a*, *qSER3b* were mapped on chromosomes 2 and 3, respectively (Tan et al., 2021). From the maintainer XieqingzaoB, *qSE7* (Zhang et al., 2018) and *qSE11* (Rahman et al., 2017) were mapped on chromosomes 7 and 11, respectively. Many QTLs for SER, such as *qPEST-5*, *qPEST-8*, *qRES-5* (Uga et al., 2003), *qRES-10* (Uga et al., 2003), *qSER-2* (Bakti and Tanaka, 2019), *qSER-3* (Bakti and Tanaka, 2019), *qPES-9*, and *qSER-1a* (Tan et al., 2020) have been found in wild rice, which is an important germplasm resource (Marathi et al., 2015; Marathi and Jena, 2015). *qES3* is a QTL of SER that has been mapped multiple times (Miyata et al., 2007) and later confirmed as the grain size gene *GS3* (Takano-Kai et al., 2011), which affects the SER by controlling the length of the stigma. Another gene, *qSTL3*, which also controls stigma length, was mapped from the indica rice Kasalath (Liu et al., 2015). However, neither of these two genes were cloned with SER as the mapping feature trait; only *LOC_Os07g15370* has been cloned using SER as the mapping feature trait (Liu et al., 2019). Unfortunately, this gene has not been confirmed to affect SER in sterile lines. Despite substantial research, most genes only stay in the mapping stage because the stigma exsertion is not only affected by genes, but also by environmental factors (e.g., temperature, moisture, wind; Yan et al., 2009). In addition, most populations used in these studies were either F_2_ populations, recombinant inbred lines, backcross inbred lines, or double haploid lines. It is difficult to exclude epistatic effects of different chromosome segments in these populations, further challenging QTL cloning of SER (Liu et al., 2015). Therefore, we presently have a poor understanding of the molecular mechanisms of SER in rice, and it is urgent to clone SER-related genes.

In our previous study, the F_2_ population was constructed using the japonica two-line male sterile line, DaS, and tropical japonica, D50, to map QTL of SER, *qPES3* on Chr3, *qPDES4* on Chr4, and *qPES12* on Chr12, and the contribution rates were 25.6, 17.86, and 16.98%, respectively (Li et al., 2017). In this study, we further constructed a near-isogenic line NIL (*qSE4*^DaS^), whose SER was significantly higher than that of D50. The *qSE4* gene was narrowed within 410.4 kb on chromosome 4 using the backcross inbred populations of BC_3_F_2_ and BC_4_F_2_. Within this region, a possible stigma exsertion-related gene was identified, and the function of the gene was preliminarily studied. These results provide a significant breakthrough for molecular level research on the SER of rice and have important value for the hybrid rice with high and stable yield in seed production.

## Materials and methods

### Population and field experiments

**Figure 1:**
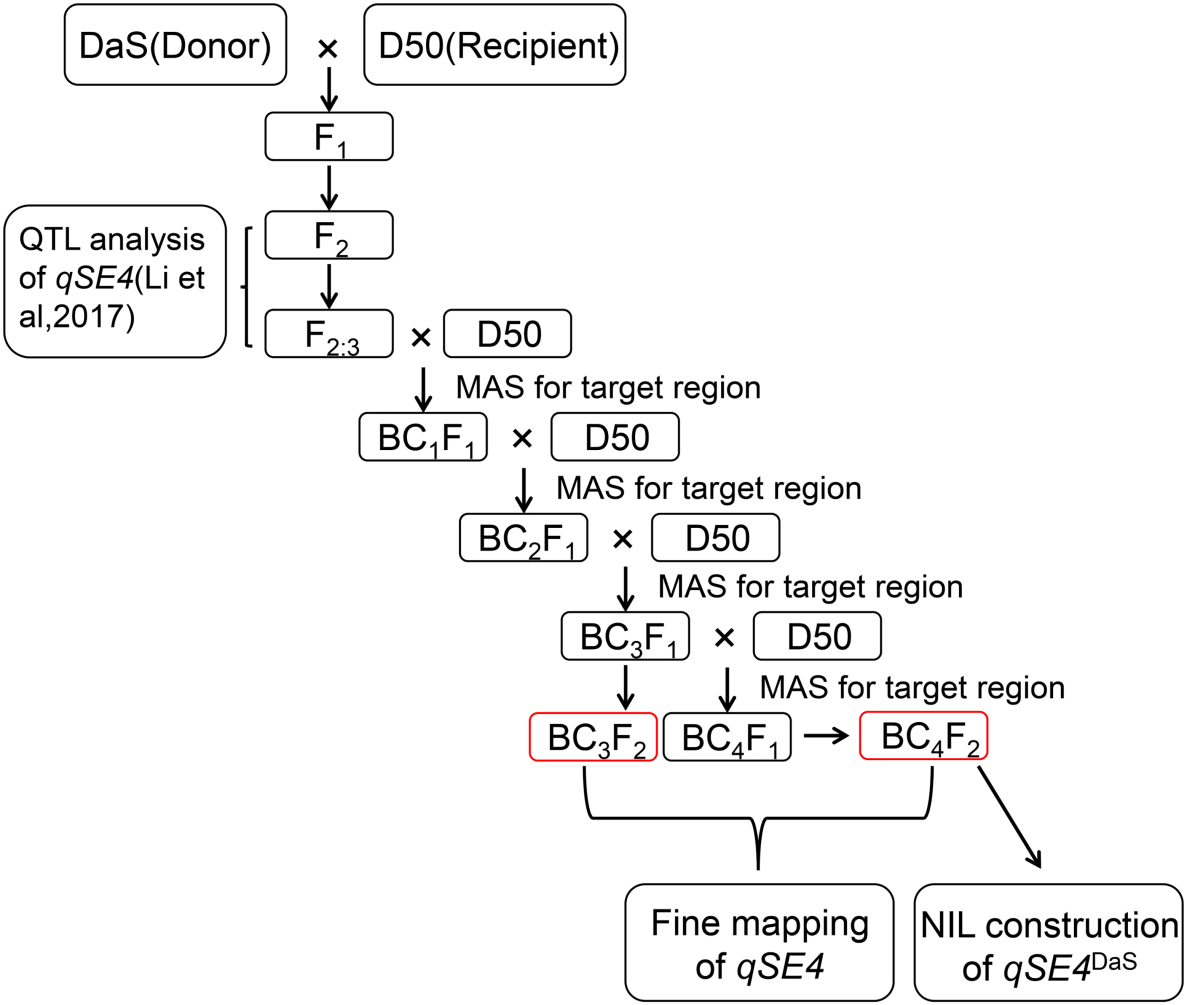
Roadmap of constructing near-isogenic lines and fine mapping populations.

The process of constructing the mapping population is shown in Figure 1. In our previous study, QTL *qSE4* was detected for a dual SER using an F_2_ population derived from a cross between D50 and DaS (Li et al., 2017). D50 is a tropical japonica with a low SER. DaS is a japonica two-line male sterile line with a high SER selected from the offspring of indica-japonica hybrids.

We fine-mapped *qSE4* by constructing a NIL with respect to *qSE4*. To this end, an F_2:3_ line with the DaS genotype in the *qSE4* region was selected to successively backcross with D50 for four generations. The SSR markers RM17157 and RM17303 were used for marker-assisted selection (MAS) of each generation among the segregating progenies. Finally, we used the BC_3_F_2_ and BC_4_F_2_ populations to map *qSE4* and selected two BC_4_F_2_ individuals as NILs. To test recovery rate of the NILs, we used 134 pairs of polymorphic SSR markers between DaS and D50, which evenly distributed on 12 rice chromosomes. It was found that the genotypes of all the markers of the two NILs were the same as those of D50 except for RM17207 and RM17227.

The *qSE4* knockout vector was constructed using the BGK03 vector of the Biogle kit and sent to Wuhan Biorun Company for the transformation of the parental DaS.

All materials were grown at the experimental field of China National Rice Research Institute or the test field in Lingshui, Hainan Province, China. Standard crop management practices were followed.

### Phenotypic evaluation

After full flowering of the rice plants, 6 main panicles were randomly selected from each line, and the single (SSE), dual (DSE), and total (TSE) SER were investigated. SSE (%) = single stigma exposed spikelets / total spikelets × 100%; DSE (%) = dual stigma exposed spikelets/total spikelets × 100%; TSE (%) = SSE + DSE.

### DNA extraction and molecular marker screening

The genomic DNA was extracted from fresh leaves using the Sodium Dodecyl Sulfate (SDS) method (Orjuela et al., 2010). Polymerase Chain Reaction (PCR) was performed in 12 μl reaction volumes containing 2 template DNA, 5 μl of 2 × T5 Super PCR Mix (PAGE; TSINGKE, Beijing, China), 1 μl of 10 μmol/μL primer pairs, and 4 μl of double distilled H_2_O. Target DNA segments were amplified with the following program, 95°C for 5 min, followed by 35 cycles of 95°C for 10 s, 55°C for 10 s, and 72°C for 10 s, and a final extension of 72°C for 8 min. The PCR products were separated by 8% non-denaturing polyacrylamide gel electrophoresis, and bands were detected using the silver staining methods described by Wang et al. (2015). Molecular markers are provided by the preservation of our laboratory.

### RNA extraction and qRT-PCR analysis

Total RNA was isolated from young panicles at the pre-heading stage (Liu et al., 2019) using Trizol reagent (Invitrogen, Carlsbad, CA, USA). Total RNA was reverse transcribed to generate cDNA using the Rever Tra Ace qPCR RT Kit (Toyobo, Osaka, Japan). Gene expression was measured by qRT-PCR using the *OsActin* (*LOC_Os03g50885*) as an internal control. PCR was performed *via* the Applied Biosystems Quant Studio 3 Real-Time PCR System (Thermo Fisher Scientific, USA). The PCR program was 95°C for 5 min, followed by 40 cycles at 95°C for 5 s, 60°C for 34 s, followed at 95°C for 15 s, 60°C for 1 min, 95°C for 15 s.

### RNA-seq analysis

Young panicles of DaS and *arf10* at the pre-heading stage were harvested and immediately frozen in liquid nitrogen. RNA-seq analysis was performed by Novogene (Beijing, China). After the RNA was reversed to double-stranded cDNA, qualified libraries were constructed, then sequenced by the Illumina NovaSeq 6,000. Differential expression analysis between the two comparisons was performed using the DESeq2 R package (1.20.0). The resulting *p*-values were adjusted using the Benjamini and Hochberg’s approach for controlling the false discovery rate. Thresholds of padj < 0.05 and |log2foldchange| > 1 were set for significant differential expression. GO enrichment analysis and KEGG enrichment analysis of differentially expressed genes were both analyzed by the cluster Profiler R package (3.8.1).

### Measurement of free IAA, ABA, and JA content

Young panicles of DaS and *arf10* at the pre-heading stage were harvested and immediately frozen in liquid nitrogen. The samples (100 mg) were resuspended with liquid nitrogen and homogenized with 400 μl of acetonitrile (50%) which contained mixed internal standards and extracted at 4°C. Then centrifuged at 12,000 rpm for 10 min. The supernatant passed through the HLB sorbent (first flow-through fraction) and then was eluted subsequently with 500 μl of acetonitrile (30%; second flow-through fraction). These two fractions were combined into the same centrifuge tube and mixed well. Finally, these solutions were injected into the LC-MS/MS system for analysis.

### Statistical analysis

One-way ANOVA was used to test the statistically significant differences among tested varieties, which was performed using SPSS 22.0 software (IBM Inc.).

## Results

### Development of the near isogenic line for *qSE4*

**Figure 2:**
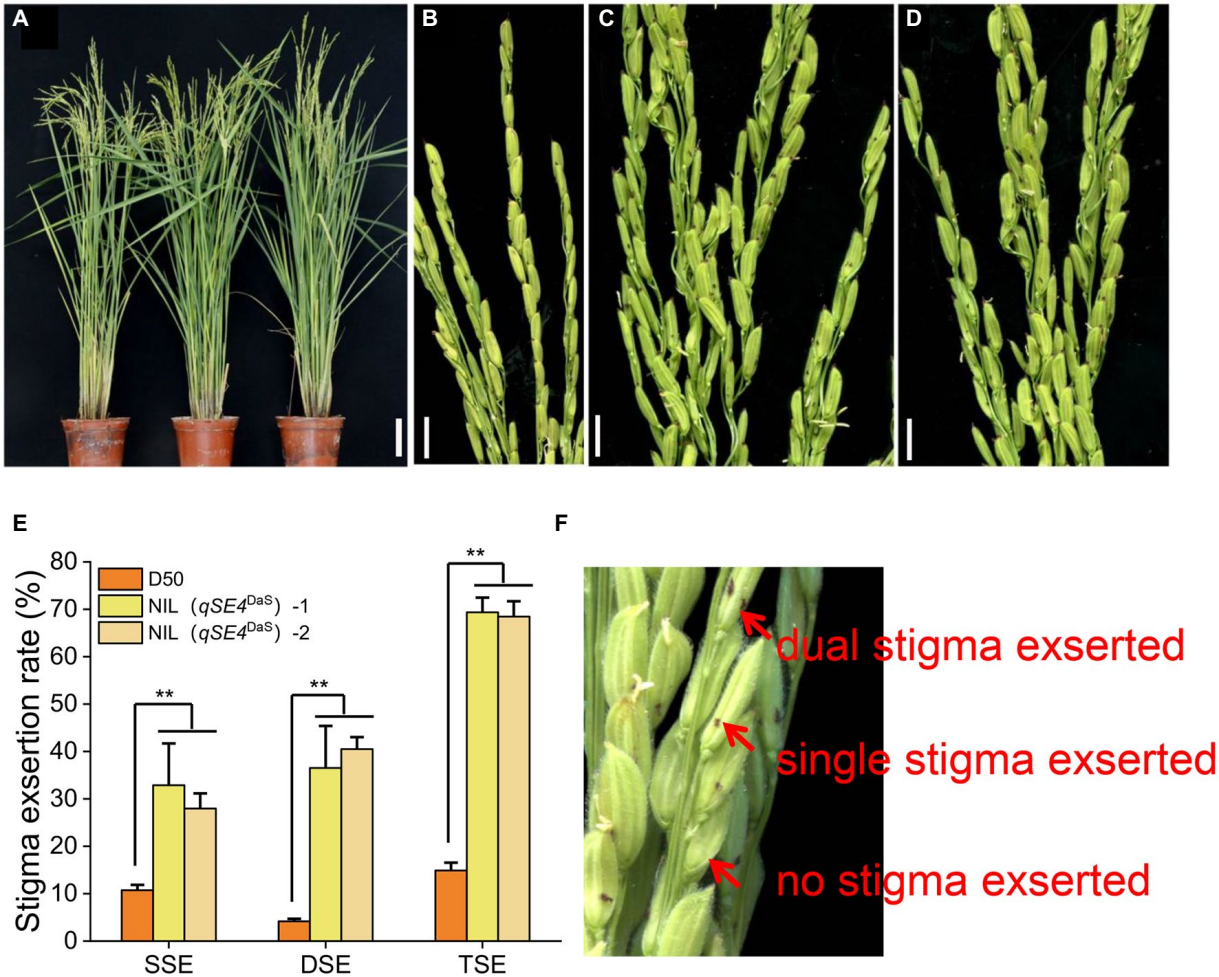
Phenotype of parental D50 and NIL. **(A)** D50, NIL (*qSE4*^DaS^)-1 and NIL (*qSE4*^DaS^)-2 from left to right during heading, bar = 10 cm. **(B)** Stigma exsertion phenotype of D50, bar = 1 cm. **(C)** Stigma exsertion phenotype of NIL (*qSE4*^DaS^)-1, bar = 1 cm. **(D)** Stigma exsertion phenotype of NIL (*qSE4*^DaS^)-2, bar = 1 cm. **(E)** Stigma exertion rate for D50, NIL (*qSE4*^DaS^)-1 and NIL (*qSE4*^DaS^)-2. SSE: single stigma exsertion rate, DSE: dual stigma exsertion rate, TSE: total stigma exsertion rate. **(F)** Stigma exsertion phenotype in rice, contains dual, single, and no stigma exsertion. The data represent the mean values ± SD (*n* = 6), ^**^p ≤ 0.01.

Based on previous research, *qSE4* was mapped between markers RM17157 and RM17303 (Li et al., 2017; Figure 2A). One line from the F_2:3_ population was selected for four rounds of backcrossing with D50, and near-isogenic lines (NILs) were used to isolate *qSE4* (Figure 1). The markers RM17157 and RM17303 were used for marker-assisted selection in the segregating progenies carrying the DaS *qSE4* allele during each backcross generation. After continuous backcrossing for four generations and selfing, the genetic background became similar to that of the recurrent parent D50, except for the substituted target segments. Two individuals with the highest recovery rate to the recurrent parent were selected among the segregating progeny, i.e., NIL (*qSE4*^DaS^)-1 and NIL (*qSE4*^DaS^)-2, which carry the homozygous allele of DaS in the region of the *qSE4*.

**Figure 3:**
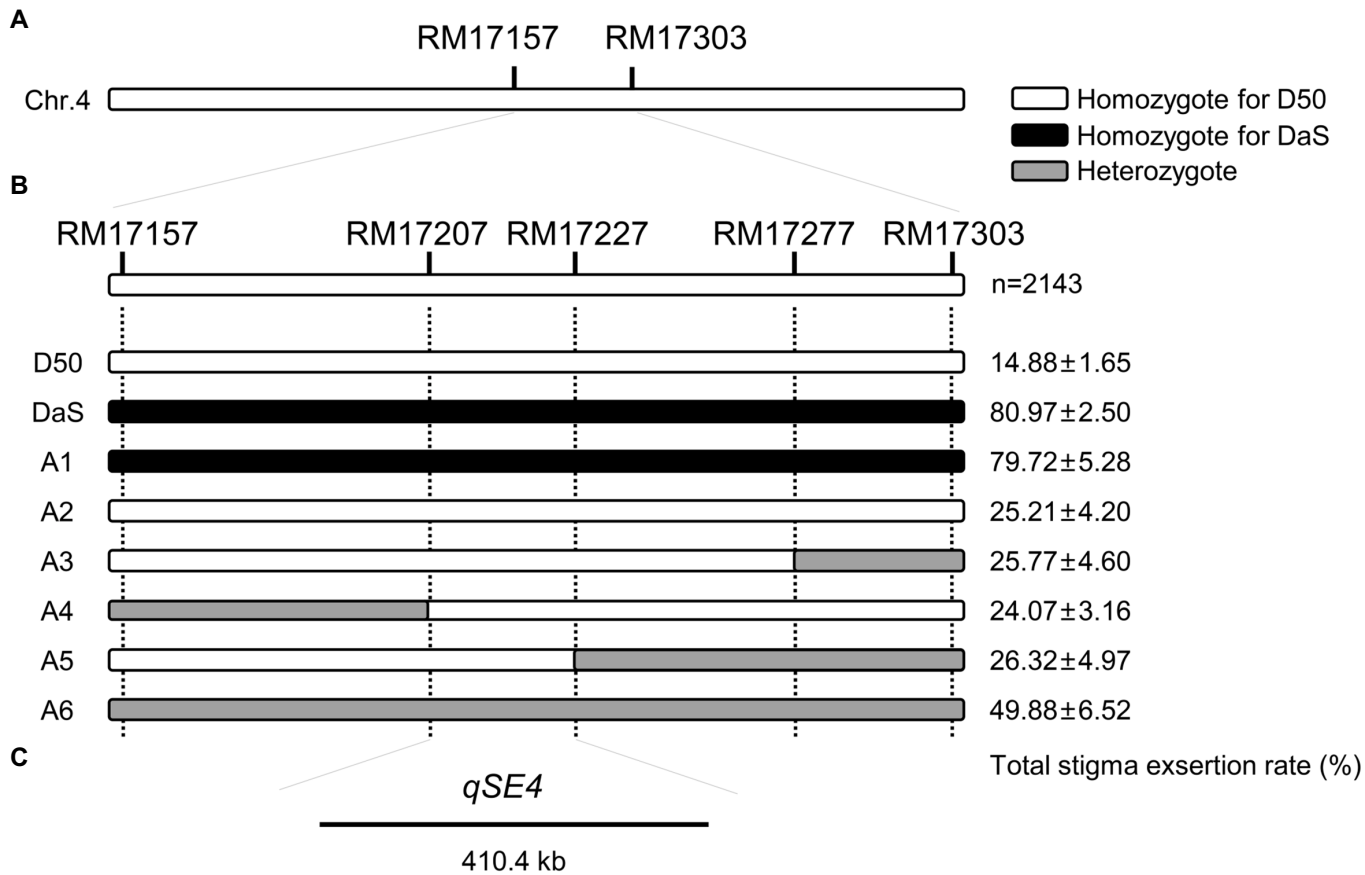
Fine mapping of *qSE4*. **(A)** *qSE4* is located between chromosome 4 markers RM17157 and RM17303. **(B)** Genotype and phenotype of D50, DaS and six recombinant individuals (A1–A6). The data represent the mean values ± SD (*n* = 6). **(C)** *qSE4* was fine mapped within 410.4 Kb between markers RM17207 and RM17227.

The NIL (*qSE4*^DaS^)-1 had increased exsertion rates of 22.14, 32.35, and 54.49% for single stigma exsertion rate (SSE), dual stigma exsertion rate (DSE), and total stigma exsertion rate (TSE), respectively, when compared to the recurrent parent D50. The NIL (*qSE4*^DaS^)-2 had increased exsertion rates of 17.25, 36.33, and 53.58% for SSE, DSE, and TSE, respectively (Table 1; Figure 3). This indicated that *qSE4* is responsible for the high stigma exsertion rate in NIL (*qSE4*^DaS^).

**Table 1:** The stigma exsertion rates of D50, NIL (*qSE4*^DaS^)-1 and NIL (*qSE4*^DaS^)-2.

Traits	D50	NIL (*qSE4*^DaS^)-1	NIL (*qSE4*^DaS^)-2
Single stigma exsertion rate (%)	10.72 ± 1.13	32.86 ± 8.84^**^	27.97 ± 3.18^**^
Dual stigma exsertion rate (%)	4.16 ± 0.55	36.51 ± 8.87^**^	40.49 ± 2.54^**^
Total stigma exsertion rate (%)	14.88 ± 1.65	69.37 ± 3.10^**^	68.46 ± 3.22^**^

NIL (qSE4^DaS^), is a near isogenic line carrying the homozygous qSE4 region from DaS under D50 genetic background. Trait values were shown as mean values ± standard deviation values. **p ≤ 0.01.

Among the random selection of 100 individuals in the BC_4_F_2_ population, the marker RM17157 was used to validate the effect of *qSE4*. The Chi-squared test revealed the phenotypic separation ratio was fitted to 1:2:1 (*X*^2^ = 3.74 < *X*^2^_0.05,2_ = 5.99), suggesting that the effect of *qSE4* is likely controlled by one genetic locus (Table 2).

**Table 2:** Marker segregation and total stigma exsertion rate in the BC_4_F_2_ population.

Marker	Numbers of plants in the BC_4_F_2_ population				Phenotyic values
	D50 homozygote	Heterozygote	DaS homozygote	*X*^2^(1:2:1)	D50 homozygote	Heterozygote	DaS homozygote
RM17215	18	59	23	3.74	20.43 ± 2.95%^A^	51.27 ± 4.33%^B^	80.06 ± 3.44%^C^

The superscript letters indicate significant differences (*p* ≤ 0.01) among the mean values within each row (Student’s *t*-test). Phenotypic values were shown as mean values ± standard deviation values.

### Fine mapping of *qSE4*

Selfing of some BC_3_F_1_ individual plants to produce BC_3_F_2_, and one heterozygote recombinant from the BC_3_F_1_ population that carried the target QTL region from DaS was backcrossed with D50 to produce a larger BC_4_F_2_ population. A total of 2,143 individuals were used for fine mapping the *qSE4* using three additional polymorphic SSR markers between DaS and D50. Five homozygous recombinant lines and one heterozygote line in the QTL region were analyzed for fine mapping (Figure 2B). The phenotypic performance of the SERs varied from 24.07 to 79.72% in the recombinant lines. The total SERs of A2, A3, A4, and A5 were similar to that of D50 and significantly lower than that of A1 which was similar to DaS. The total SER of heterozygous A6 was between that of the two parents (Figure 2B). Based on genetic and phenotypic analysis, the location of *qSE4* was narrowed to a 410.4-kb region between the RM17207 and RM17227 (Figure 2C).

### Candidate gene analysis of *qSE4*

**Figure 4:**
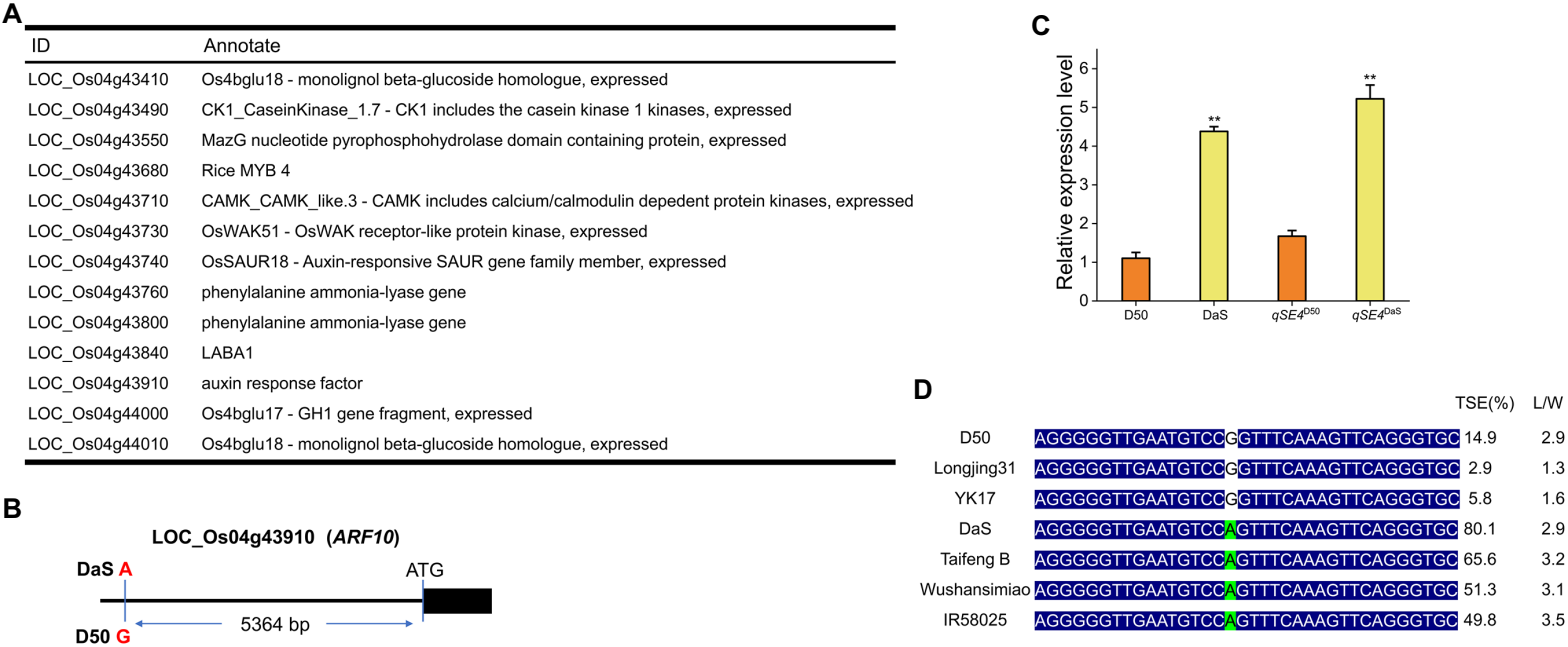
Identification of candidate genes for *qSE4*. **(A)** Thirteen candidate genes for *qSE4* (http://rice.plantbiology.msu.edu/, MSU- version_7.0). **(B)** At 5364 bp upstream of the ATG initiation codon of the gene *ARF10*, there was one base different between the two parental DaS and D50, DaS was A, and D50 was G. **(C)** Relative expression of *ARF10* in DaS, D50, RIL (*qSE4*^D50^) and RIL (*qSE4*^DaS^). **(D)** The relationship between the haplotype in the 5,364 base upstream of ATG and stigma exsertion phenotype in different rice varieties. TSE: total stigma exsertion rate; L/W: length/width. The data represent the mean values ± SD (*n* = 6), ^**^p ≤ 0.01.

**Figure 5:**
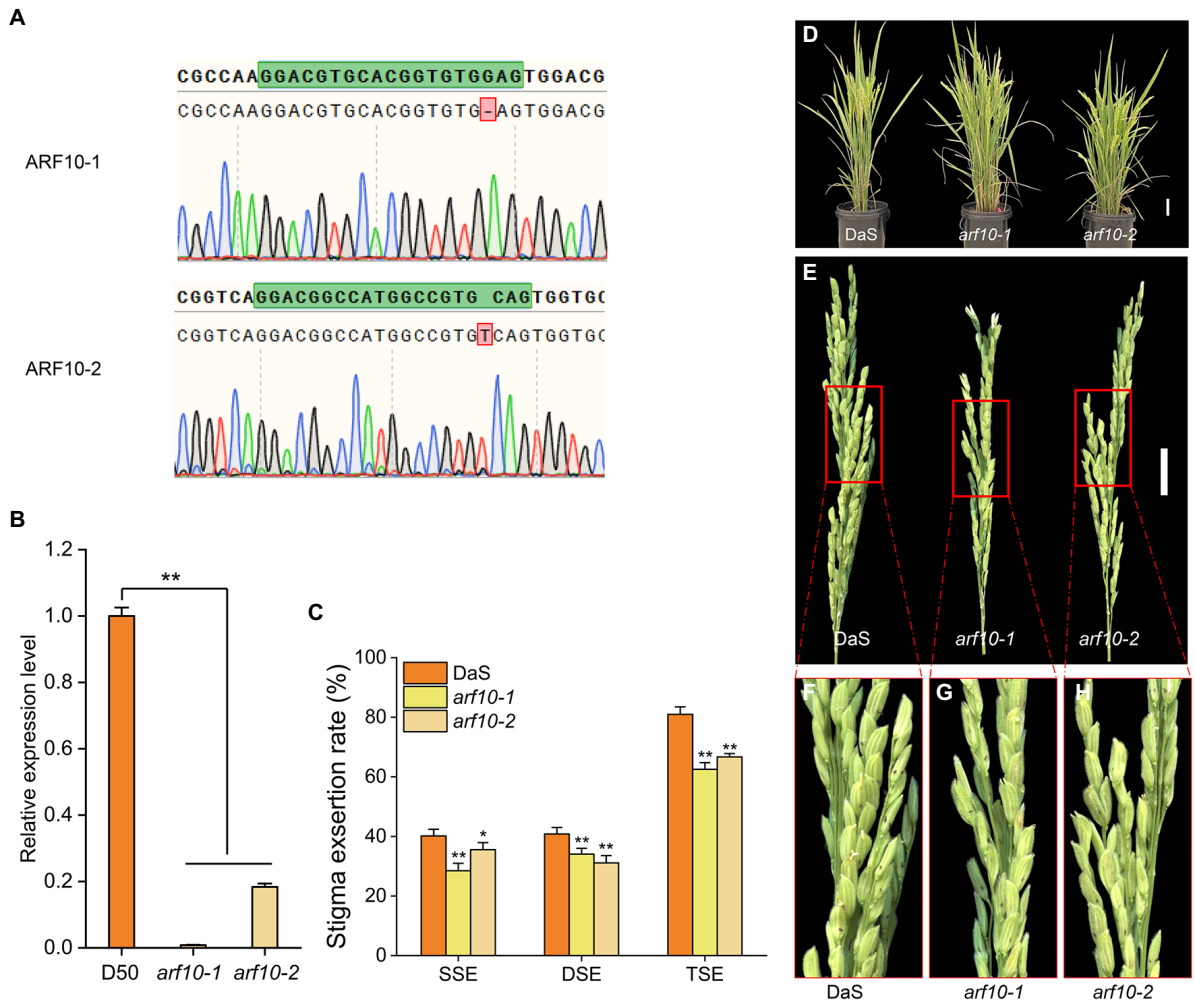
Stigma exsertion phenotype of knockout line *arf10* and the wild type DaS. **(A)** *ARF10* mutant sites of two knockout lines *arf10*. **(B)** Relative expression levels of *ARF10* in two knockout lines and the wild type DaS (*n* = 3). **(C)** Plant stigma exsertion phenotypes of the two knockout lines and the wild type DaS at the heading stage. **(D)** Stigma exsertion phenotypes at heading in the two knockout lines and the wild DaS, bar = 10 cm. **(E)** Stigma exsertion rate of two knockout lines and the wild type DaS at heading stage, bar = 5 cm. The data represent the mean values ± SD (*n* = 6), ^*^p ≤ 0.05, ^**^p ≤ 0.01.

After removing unknown retrotransposons, transposons, and putative genes (Li et al., 2021) within the 410.4-kb region, 13 genes remained (Figure 4A) according to the Rice Genome Annotation Database (rice.plantbiology.msu.edu/, MSU-version_7.0). Sequencing analysis found that the two parents had one base difference in the promoter region of *LOC_Os04g43910* (A for DaS and G for D50), but no difference in the coding region (Figure 4B). Gene expression analysis showed that the expression level of *LOC_Os04g43910* in parental DaS was significantly higher than in D50. The expression level of lines carrying the DaS allele in progenies was also significantly higher than that carrying the D50 allele (Figure 4C). The differences of *LOC_Os04g43910* among different cultivars were further analyzed, and it was found that the cultivars of haplotype G were mostly wide-grained cultivars with lower SERs. The haplotype A cultivars were mostly slender grains with higher SERs (Figure 4D). Therefore, *LOC_Os04g43910* is predicted as the candidate gene. Further, *LOC_Os04g43910* (*ARF10*) was knocked out in the DaS background using CRISPR/Cas9 technology, two mutants which named *arf10-1* and *arf10-2* were obtained. The *arf10-1* and *arf10-2* mutant plants contained 1-bp deletion and 1-bp insertion, respectively, in the exon of *ARF10* (Figure 5A). These two mutations caused the premature appearance of stop codons in the *ARF10* gene, resulting in a significant decrease in the expression of *ARF10* (Figure 5B). The SERs were significantly reduced in both knockout lines compared to the wild type (Figures 5C–H; Table 3). These results further confirmed that *ARF10* was most likely to be the causal gene for the *qSE4* influencing the SER in rice.

**Table 3:** The stigma exsertion rates of DaS, *arf10*-1 and *arf10*-2.

Traits	DaS	*arf10*-1	*arf10*-2
Single stigma exsertion rate (%)	40.15 ± 2.24	28.48 ± 2.48^**^	35.56 ± 2.37^*^
Dual stigma exsertion rate (%)	40.82 ± 2.17	34.05 ± 1.95^**^	31.12 ± 2.45^**^
Total stigma exsertion rate (%)	80.97 ± 2.50	62.54 ± 2.22^**^	66.68 ± 1.14^**^

arf10 is a knockout line of LOC_Os04g43910. Trait values were shown as mean values ± standard deviation values.^*^p ≤ 0.05; ^**^p ≤ 0.01.

### Transcriptome analysis of *ARF10* knockout plants

**Figure 6:**
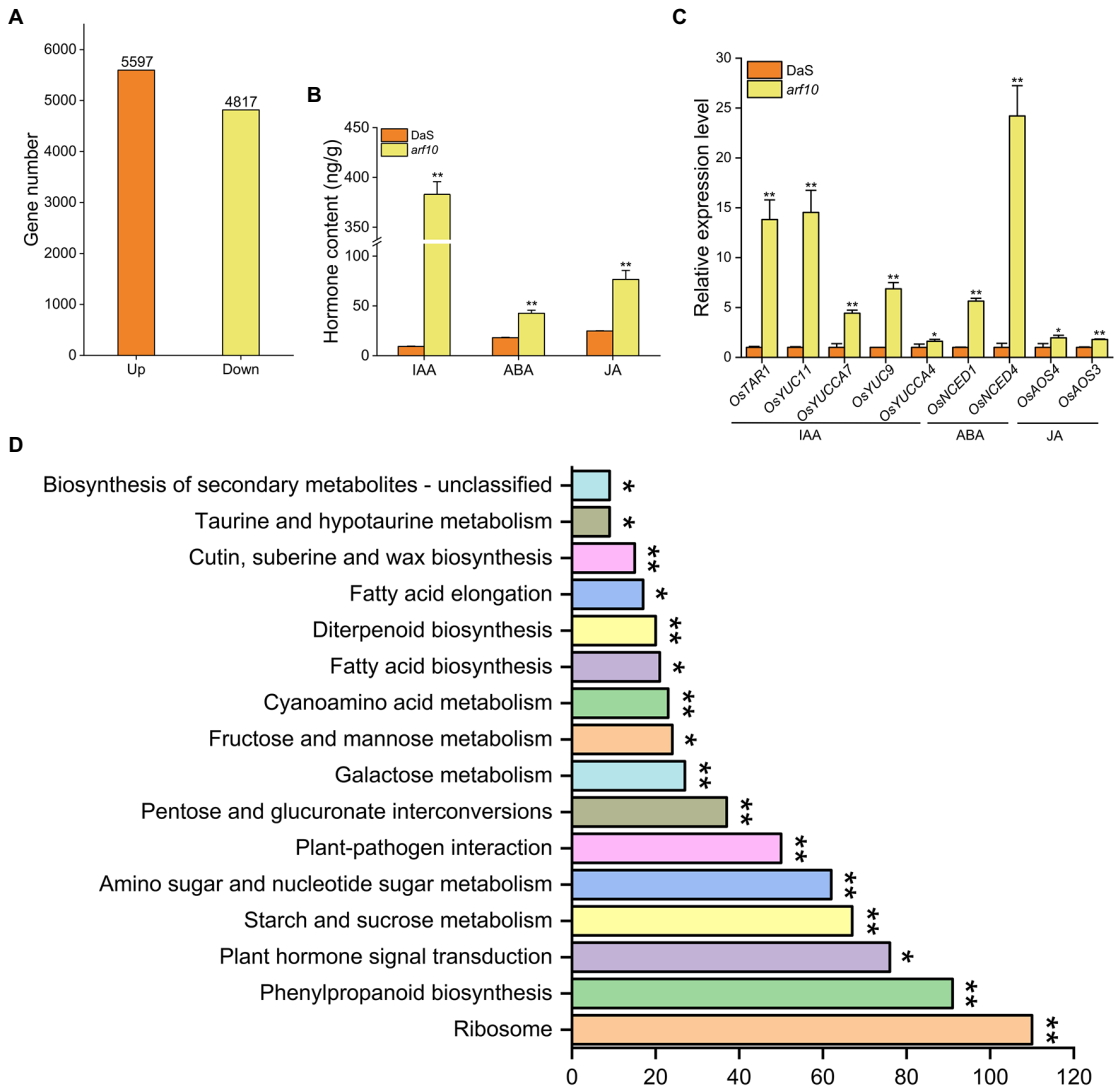
Transcriptome analysis of knockout lines *arf10* and DaS. **(A)** Number of up- and down-regulated genes (padj <0.05) in young panicle at the pre-heading stage of *arf10* compared with DaS. **(B)** KEGG pathway enrichment analysis of DEGs. **(C)** Contents of auxin, abscisic acid, and jasmonic acid in *arf10* and DaS at the pre-heading stage. **(D)** Relative expression of auxin, abscisic acid, and jasmonic acid synthesis genes of *arf10* and DaS at the pre-heading stage. The data represent the mean values ± SD (*n* = 3), ^*^p ≤ 0.05 and ^**^p ≤ 0.01.

RNA-sequencing (RNA-seq) was conducted to show the transcriptomic variation in the young panicle between the wild-type DaS and the mutant *arf10* (Figure 6). A total of 10,414 differentially expressed genes (DEGs) were detected (padj <0.05), of which 53.7% (5,597 genes) were up-regulated and 46.3% (4,817 genes) were down-regulated in the *arf10* plant compared with the expression levels in DaS plant (Figure 6A; Supplementary Figure 1). We randomly selected 8 genes to analyze their expression levels *via* qRT-PCR. The results were consistent with the results of the transcriptome (Supplementary Figure 2). GO analysis reveals DEGs enrichment in biological processes, cellular components, and molecular functions. Metabolic process, biochemical reaction, and biosynthetic process represent 41.32, 16.51, and 11.85% of total DEGs, respectively, and were the largest subcategories in the biological process. Ribosome, cell periphery, and external encapsulating structure represent 30.17, 21.55, and 14.08% of total DEGs, respectively, and were the largest subcategories in the cellular component. Enzyme activity, binding, and transmembrane transporter activity represent 43.67, 28.85, and 3.90% of total DEGs, respectively, and were the largest subcategories in the molecular function (Supplementary Figure 3). KEGG pathway analysis showed that ribosome, phenylpropanoid biosynthesis, and plant hormone signal transduction were the most significantly enriched pathways (padj <0.05) of DEGs (Figure 6D).

Since *ARF10* is an auxin-responsive factor, we detected the hormone content of DaS and *arf10* and analyzed the expression of hormone synthesis and signaling-related genes. Compared with wild-type DaS, the content of IAA, ABA, and JA in the knockout line *arf10* was significantly increased (Figure 6B). It was further found that the genes *OsTAR1*, *OsYUC11*, *OsYUCCA7*, *OsYUC9,* and *OsYUCCA4* for the IAA synthesis pathway; *OsNCED1* and *OsNCED4* for the ABA synthesis pathway; *OsAOS3* and *OsAOS4* for the JA synthesis pathway were significantly higher in *arf10* than in DaS (Figure 6C). In addition, the genes of IAA, ABA, and JA signaling pathways were also expressed significantly differently in *arf10* and DaS (Supplementary Figure 4). These results suggested that hormones are key factors affecting the SER.

## Discussion

The application of hybrid rice has greatly improved rice yields, but the low efficiency of hybrid seed production limits its promotion in Southeast Asia (Xie, 2008). The increase of stigma exsertion can increase hybrid seed production and promote the commercialization of hybrid rice (Zhou et al., 2017). In the past two decades, dozens of QTLs of the SER have been mapped and found to be distributed on 12 chromosomes in rice. However, only two were located on chromosome 4. One is *qSER4*, a QTL that reduces the SER identified from wild rice ‘W0120’, which is close to marker C5-indel3632 (physical location Chr4:5050622; Bakti and Tanaka, 2019). The other is *PES4*, which was identified from the upland rice maintainer Huhan1B, which was located between the markers RM6909 and RM1113 on chromosome 4. In this study, we used DaS, a japonica two-line sterile line with a high SER, and D50, a tropical japonica rice with a low SER, to construct a backcross inbred mapping population and mapped the major QTL: *qSE4*, which controls the SER, between the markers RM17207 and RM17227 on Chromosome 4 (Figure 2C). *qSE4* is far from *qSER4*, but within the same interval of RM6909 and RM1113 as *PES4*. Thus, *qSE4* is a QTL that controls SERs and can be detected reproducibly across different germplasm resources.

Because it is beneficial to the application of heterosis, stigma exsertion is a research hotspot for many crops. So far, only a few genes related to stigma exsertion have been cloned from limited crops. In tomato, the transcription factor *Style2.1* can regulate cell elongation, and the difference in the promoter region of *Style2.1* among different varieties leads to different levels of gene expression, determining whether the tomato stigma is exposed or inserted (Chen et al., 2007). This is different from the *GS3* gene in rice, where mutations in *GS3* promote an increase in the number of cells, leading to elongation of the stigma (Takano-Kai et al., 2011). The zinc-finger transcription factor, *SE3.1* in tomatoes, shows another newly discovered regulatory mechanism. *SE3.1* controls stigma exsertion, which is not related to its expression level but is related to its gene function. The loss of *SE3.1* function promotes tomato stigma retraction, while *Style2.1* and *SE3.1* are key genes that regulate the two-step process of stigma from exsertion to insert during tomato domestication (Shang et al., 2021). Recently, there was a major breakthrough in the study of stigma exsertion in legumes. It was found that *SGE1* controls the stigma exsertion of *Medicago truncatula* by regulating the secretion of keratin and wax of flowers (Zhu et al., 2020). In this study, *qSE4* was fine-mapped within 410.4 kb, and 13 candidate genes were identified *via* bioinformatics analysis (Figure 4A). qRT-PCR and sequencing analysis showed that a nucleotide change at 5364 bp of the promoter of *LOC_Os04g43910* (*ARF10*) could affect the expression of this gene, thereby affecting the stigma exsertion rate (Figures 4B,C). Haplotype analysis further indicated that 5364^G^ was associated with low stigma exsertion and 5364^A^ was associated with high stigma exsertion (Figure 4D). The way *ARF10* regulates stigma exsertion is similar to that of *Style2.1*, and the stigma exsertion is determined by the change in expression level, so *ARF10* is likely to be a candidate gene of *qSE4*. We further validated its function by knocking out *ARF10* in the DaS background. The expressions of *ARF10* in the two knockout lines was significantly lower than of the wild type (Figure 5B), and the SER was also significantly lower than that of the wild type (Figure 5C). These results point to *ARF10* as a target gene of *qSE4*.

Auxin response factors (*ARF*s) are a class of transcription factors that regulate the response to plant auxins (Wang et al., 2007). Twenty-five *ARF*s family member genes were found in rice, which plays important functions in rice disease resistance (Qin et al., 2020; Zhang et al., 2020; Zhao et al., 2020), root growth (Qi et al., 2012; Shen et al., 2013; Shao et al., 2019), and plant morphology (Attia et al., 2009; Sakamoto et al., 2013; Zhang et al., 2015; Huang et al., 2016, 2021; Chen et al., 2018; Liu et al., 2018). *ARF25* may regulate the expression of the auxin synthesis gene *OsYUCCA*s to affect the auxin content of rice roots (Qi et al., 2012), while *ARF16* may regulate auxin redistribution (Shen et al., 2015). Compared with wild DaS, the auxin content and five auxin synthesis genes in *arf10* increased significantly (Figures 6B,C), suggesting that *ARF10* may also regulate the expression of auxin synthesis genes to affect the auxin content in rice young panicles. Auxin has a dual effect on plant growth, low concentrations promote growth and high concentrations inhibits growth, therefore, the high concentration of auxin in *arf10* may be the reason for its reduced SER. Consistent with this notion, low concentrations of IAA were found in tobacco to promote stigma growth while high concentrations inhibited stigma growth (Chen and Zhao, 2008).

Various biochemical processes in plants are regulated by hormone crosstalk. Auxin also performs some functions in conjunction with other hormones. Auxin and abscisic acid jointly control seed dormancy in *Arabidopsis thaliana* (Liu et al., 2013), and together with brassinolide regulate plant height and leaf angle (Liu et al., 2018). The contents of ABA and JA in *arf10* were significantly higher than those of the wild type (Figure 6B), and their synthetic genes also increased significantly (Figure 6C), suggesting that *ARF10* may be involved in the synthesis of ABA and JA. KEGG analysis showed that the differential genes were enriched in the hormone signaling pathway (Figure 6D; Supplementary Figure 4). These results all indicated that IAA, ABA, and JA may jointly regulate the degree of stigma exsertion in rice.

The development of NILs is an effective strategy for studying QTLs as the interference of the noise from the background is significantly reduced (Ding et al., 2011). In our study, NIL of the *qSE4* carrying DaS allele in the D50 background significantly increased the stigma exsertion rate compared with the recurrent parent, D50 (Figure 3E), suggesting that *qSE4* is responsible for the increased SER in NIL. This not only provides the basis for the subsequent cloning of the *qSE4* target gene but also provides molecular markers linked to the *qSE4* target gene. Molecular markers can be applied to gene pyramid breeding in rice with a high SER. Cultivating rice male sterile lines with a high SER can increase the yield of hybrid rice seed production, reduce the production cost of hybrid rice, and promote hybrid rice development with health and sustainability.

## Data availability statement

The datasets presented in this study can be found in online repositories. The names of the repository/repositories and accession number(s) can be found at: https://www.ncbi.nlm.nih.gov/bioproject/PRJNA850518.

## Author contributions

HP and SZ designed experiments. GN, WY, CW, TS, AR, WX, HS, TS, SG, JG, XL and WL performed experiments. GN analyzed data and compiled figures. GN wrote the manuscript. SZ edited the final manuscript. All authors contributed to the article and approved the submitted version.

## Funding

This research was financially supported by the China Natural Science Foundation (no. 31871597), Zhejiang Science and Technology Major Program on Agricultural New Variety Breeding (2021C02063-2), and the Key Research and Development Program of China National Rice Research Institute (CNRRI-2020-02).

## Conflict of interest

The authors declare that the research was conducted in the absence of any commercial or financial relationships that could be construed as a potential conflict of interest.

## Publisher’s note

All claims expressed in this article are solely those of the authors and do not necessarily represent those of their affiliated organizations, or those of the publisher, the editors and the reviewers. Any product that may be evaluated in this article, or claim that may be made by its manufacturer, is not guaranteed or endorsed by the publisher.

## Supplementary materials

The Supplementary materials for this article can be found online at: https://www.frontiersin.org/articles/10.3389/fpls.2022.959859/full#supplementary-material
