# Supplementary material for: Fine mapping and target gene identification of qSE4, a QTL for stigma exsertion rate in rice (Oryza sativa L.)
Source: Front Plant Sci. 2022 Jul 18;13:959859. doi: 10.3389/fpls.2022.959859 (PMC9341389; doi:10.3389/fpls.2022.959859)
Supplement: Supplementary file 1 [file Data_Sheet_1.docx]

Supplementary Material

# Supplementary Figures


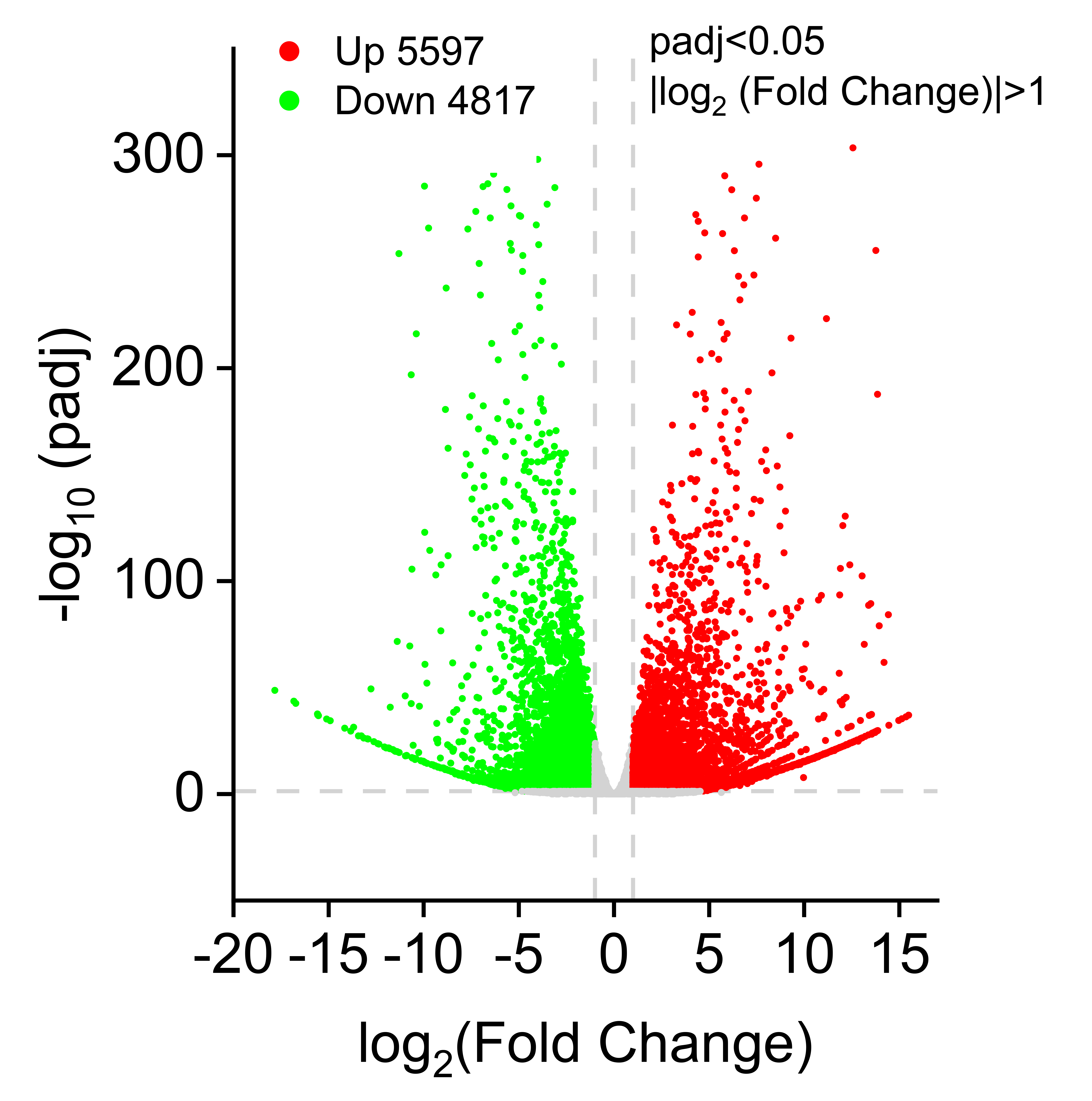


**Supplementary Figure 1.**Volcano plot of differentially expressed genes
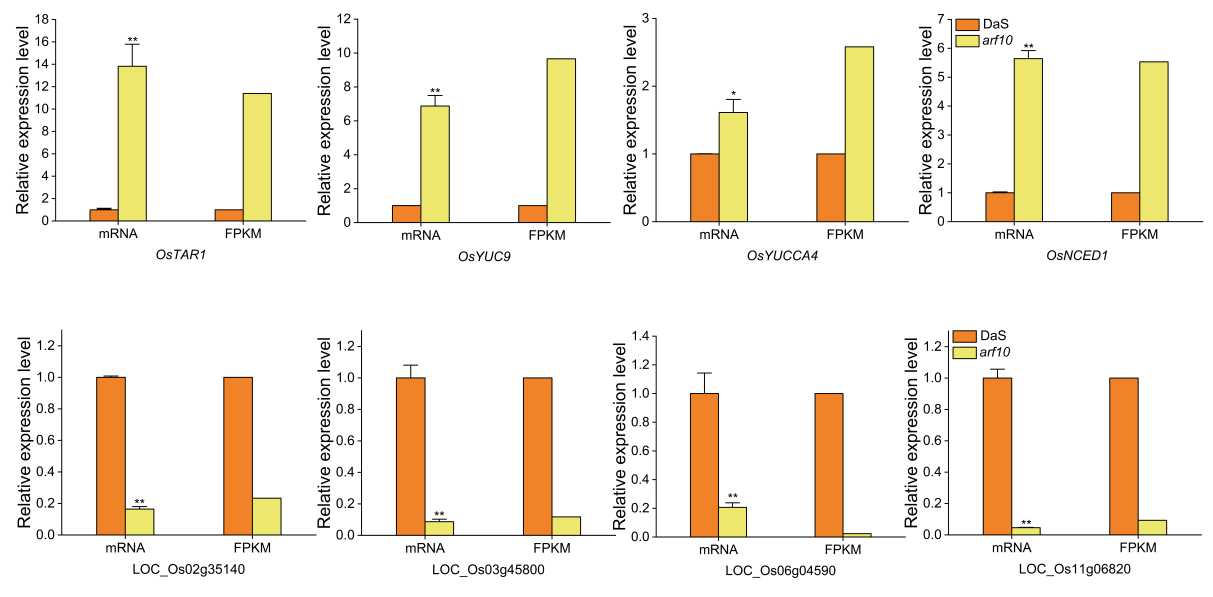


**Supplementary Figure 2.** Eight randomly selected genes for qRT-PCR comparison with transcriptome data. The data represent the mean values ± SD (n = 3), *P ≤ 0.05, **P ≤ 0.01.


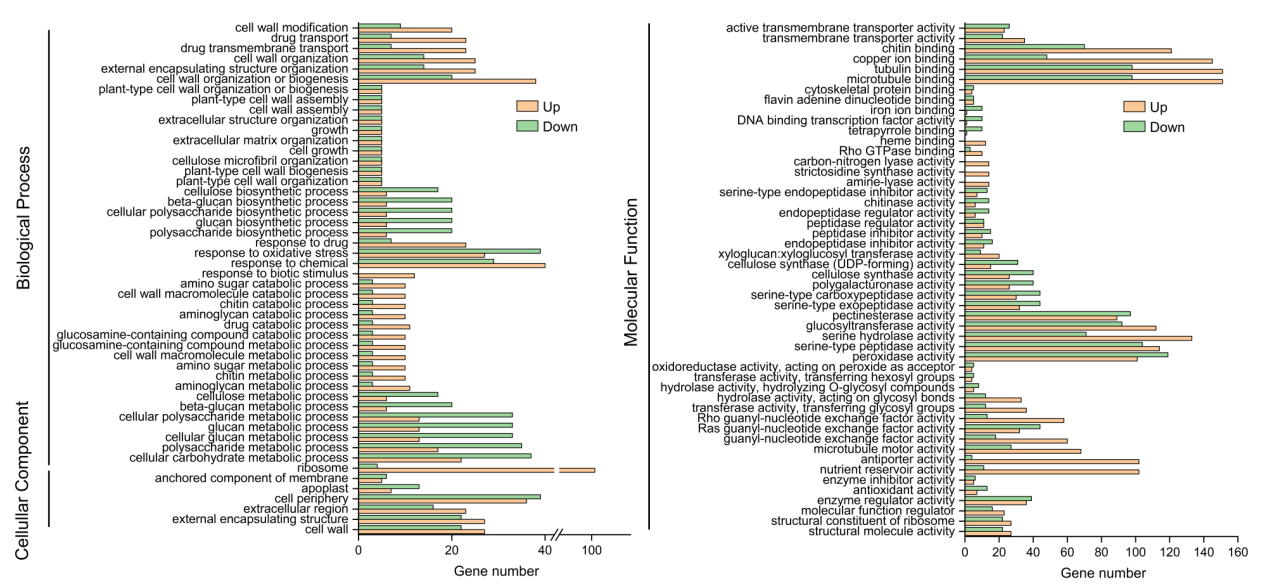


**Supplementary Figure 3.** GO analysis of DEGs (padj < 0.05), divided into biological processes, cellular composition, and molecular functions.


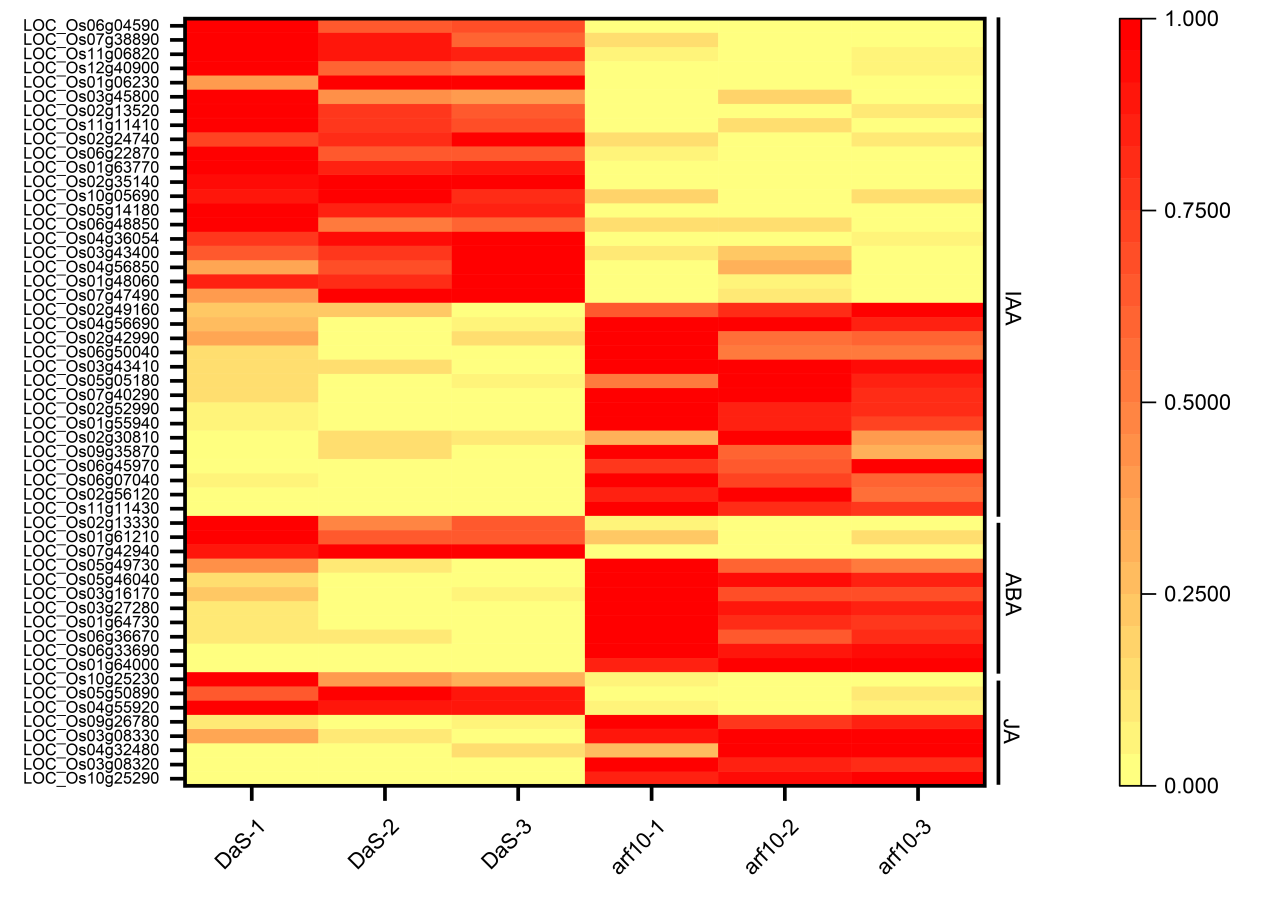


**Supplementary Figure 4.** DEGs (padj < 0.05) of signal transduction genes for auxin, abscisic acid, and jasmonic acid in knockout lines *arf10* and DaS

# Supplementary Tables

**Supplementary Table 1.** Primers used in this study

| **Primers** | **Sequence (5’-3’)** | **Description** |
| --- | --- | --- |
| RM17157-F | ACCGCTCGTCACAACTCACACG | Mapping |
| RM17157-R  RM17207-F  RM17207-R  RM17227-F  RM17227-R  RM17277-F  RM17277-R  RM17303-F  RM17303-R | GTCTTCAGCCGCCACAAGAAGG  GCATCAAGAGAGATCTGCATACCC  ATCCTCCATTTCGCATGTATGG  GCTACGGCGCTGAAATACTCC  GTCCAAACATCCAATGTGAGTCC  GGTCTCTCCTGCCTTGACACTCC  AAGGTGGCATTGACTCACGAACC  TAGCGATTGGATGGAGGCTGAGG  GGTCGCCTCGCCATTAGTTACG |  |
| CRI-*ARF10*-1-F | TGTGTGggacgtgcacggtgtggag | Gene knockout |
| CRI-*ARF10*-1-R  CRI-*ARF10*-2-F  CRI-*ARF10*-2-R | AAACctccacaccgtgcacgtccCA  TGTGTGGGACGGCCATGGCCGTGCAG  AAACCTGCACGGCCATGGCCGTCCCA |  |
| Actin.qRT-F | TCTGGCATCACACCTTCTACA | qRT-PCR |
| Actin.qRT-R | GGAAGGCTGGAAGAGGAC |  |
| *ARF10*.qRT-F | GAAGATCGACGACACCAAGC |  |
| *ARF10*.qRT-R | GCTGATGTTGTTGCCTCCAA |  |
| *OsTAR1*.qRT-F | ATGCTCTTCACCGTCTCCAA |  |
| *OsTAR1*.qRT-R | CGATGGTGTTGAGCTCGATG |  |
| *OsYUC9*.qRT-F | AAGAGTGATGACGGGCTGAT |  |
| *OsYUC9*.qRT-R | CGTAGATTCCCCTCCTCACC |  |
| *OsYUCCA4*.qRT-F | GAACATGGCGTGGAGTTTGT |  |
| *OsYUCCA4*.qRT-R | CACGCCTTGAGAAACCAACA |  |
| *OsNCED1*.qRT-F | AACTTCGACTTCCCCGTGAT |  |
| *OsNCED1*.qRT-R | CACCAAGTACCCGTCATCCT |  |
| LOC_Os02g35140.qRT-F | GCAAGGAATGGCAGTTGGAA |  |
| LOC_Os02g35140.qRT-R | TCATCCCCAACCAGCATCAT |  |
| LOC_Os03g45800.qRT-F | AGGGACGAAGAAGAAGGCG |  |
| LOC_Os03g45800.qRT-R | CGTAGCCGTACTCCTGCG |  |
| LOC_Os06g04590.qRT-F | GAGGAGGAGTTCGGCTTCG |  |
| LOC_Os06g04590.qRT-R  LOC_Os11g06820.qRT-F  LOC_Os11g06820.qRT-R | AGGAGTAGTGGAGGGAGGAG  ACTGCACCTTCCTCCTCTTC  TAGTTGTGGAAGGACGGGAC |  |
